# Internally driven large‐scale changes in the size of Saturn's magnetosphere

**DOI:** 10.1002/2015JA021290

**Published:** 2015-09-10

**Authors:** N. M. Pilkington, N. Achilleos, C. S. Arridge, P. Guio, A. Masters, L. C. Ray, N. Sergis, M. F. Thomsen, A. J. Coates, M. K. Dougherty

**Affiliations:** ^1^ Atmospheric Physics Laboratory, Department of Physics and AstronomyUniversity College LondonLondonUK; ^2^ The Centre for Planetary SciencesUCL/BirkbeckLondonUK; ^3^ Physics DepartmentLancaster UniversityLancasterUK; ^4^ Blackett LaboratoryImperial College LondonLondonUK; ^5^ Academy of AthensOffice of Space Research and TechnologyAthensGreece; ^6^ Planetary Science InstituteTucsonArizonaUSA; ^7^ Mullard Space Science Laboratory, Department of Space and Climate PhysicsUniversity College LondonDorkingUK

**Keywords:** Saturn, magnetosphere, solar wind, plasma, magnetopause

## Abstract

Saturn's magnetic field acts as an obstacle to solar wind flow, deflecting plasma around the planet and forming a cavity known as the magnetosphere. The magnetopause defines the boundary between the planetary and solar dominated regimes, and so is strongly influenced by the variable nature of pressure sources both outside and within. Following from Pilkington et al. (2014), crossings of the magnetopause are identified using 7 years of magnetic field and particle data from the Cassini spacecraft and providing unprecedented spatial coverage of the magnetopause boundary. These observations reveal a dynamical interaction where, in addition to the external influence of the solar wind dynamic pressure, internal drivers, and hot plasma dynamics in particular can take almost complete control of the system's dayside shape and size, essentially defying the solar wind conditions. The magnetopause can move by up to 10–15 planetary radii at constant solar wind dynamic pressure, corresponding to relatively “plasma‐loaded” or “plasma‐depleted” states, defined in terms of the internal suprathermal plasma pressure.

## Key Points

- Internal plasma imparts a strong degree of variability to the size of Saturn's magnetosphere
- Including this effect in empirical models improves accuracy substantially
- Solar wind conditions alone are not enough to infer magnetospheric size

## 1 Introduction

The interaction between the solar wind and the magnetic field of a planetary body gives rise to the formation of a magnetosphere, which encloses the planet and shields it from direct bombardment by plasma of solar origin. The magnetopause is the boundary that separates these populations and it forms where the solar wind dynamic pressure is balanced by internal pressure sources when the boundary is stationary. In reality, however, the pressure on either side of the boundary is highly dynamic and the magnetopause is in almost continual acceleration [e.g., *Kaufmann and Konradi*, 1969].

At Earth, the principal internal pressure source is the magnetic pressure. Saturn differs in this regard. Measurements made by *Voyagers* 1 and 2 found that energetic plasma is ubiquitous within Saturn's magnetosphere [*Krimigis et al.*, 1982, 1983]. Later, early in the Cassini mission, it was found that Enceladus ejects plumes of water group molecules into Saturn's magnetosphere [e.g., *Dougherty et al.*, 2006; *Porco et al.*, 2006]. A small fraction of these are ionized into a plasma, and this can greatly influence the dynamics that drive the magnetosphere. Estimates vary substantially but *Bagenal and Delamere* [2011] find that the plasma source rates lie between 12 and 250 kg *s* ^−1^. Similarly, Io is a large source of plasma within Jupiter's magnetosphere with typical plasma source rates exceeding those of Enceladus in absolute terms by at least an order of magnitude. However, *Vasyliunas* [2008] showed that Enceladus may be a more significant plasma source to Saturn's magnetosphere than Io is to Jupiter's magnetosphere because, in relative terms, it may cause flux tubes to become more heavily loaded with mass and hence perturb Saturn's magnetic field more strongly.

At Saturn's magnetopause, the pressure associated with the suprathermal component of this plasma is of the same order as the magnetic pressure and acts to inflate the magnetosphere, significantly increasing its size beyond what would be expected of the magnetic pressure alone. *Sergis et al.* [2007, 2009] found that the plasma sheet extends all the way out to the dayside magnetopause boundary and that the plasma *β* at Saturn (the ratio of plasma to magnetic pressure) for ions with energies greater than 3 keV, at radial distances concurrent with the magnetopause, varies between ∼10^−2^ and 10^1^. Plasma dynamics are thus likely to have a significant impact on the size and shape of the Kronian magnetopause due to the highly variable nature of *β* just inside.

Previous empirical studies have treated the solar wind dynamic pressure as the primary source of variability in the location of the magnetopause. However, magnetohydrodynamic (MHD) studies of the Kronian magnetosphere [e.g., *Zieger et al.*, 2010] found that internal plasma dynamics can change the geometry of Saturn's magnetopause significantly under conditions when the solar wind pressure is low. Moreover, no steady state magnetopause boundary is obtained in these simulations under low solar dynamic pressure conditions. Here it will be shown that internal plasma dynamics imparts a similar degree of variability to the location of Saturn's magnetopause as does variability in the solar wind pressure. In addition to this aspect, previous studies are expanded upon by including high‐latitude observations of Saturn's magnetopause in both hemispheres and near‐equatorial observations of both the morning and the evening sectors, providing much greater coverage of the dayside magnetopause. Furthermore, a more sophisticated fitting routine is used and a new method of calculating the perpendicular distance between the crossing and the model surface near‐exactly is presented in order to fit an empirical model to these data more accurately. A more realistic estimate for the thermal ion pressure at the magnetopause is also calculated.

In section 2, previous empirical models of Saturn's magnetopause and the improvements made in this study are outlined, in section section 3 the in situ magnetopause observations are discussed, and in section 4.1 the results of fitting the model to the Cassini data are presented and discussed. In section 4.2, a substantial enhancement is made to the empirical model in order to address a major shortcoming in its application to magnetospheres with significant internal plasma sources. These results are further discussed and summarized in section 5.

## 2 The Model

### 2.1 Previous Work

**Figure 1:**
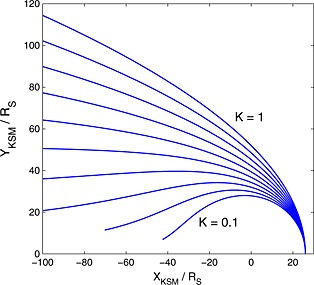
This figure demonstrates how varying the flaring parameter, *K*, changes the magnetopause geometry, *K* is varied between 0.1 (innermost line) and 1.0 (outermost line) in this plot [after *Shue et al.*, 1997].

The *Shue et al.* [1997] empirical shape model was originally devised to model the terrestrial magnetopause,

(1) $$r=r_{0}{\left(\frac{2}{1+\cos\theta }\right)}^{K}$$

(2) $$r_{0}=a_{1}D_{P}^{-a_{2}}$$

(3) $$K=a_{3}+a_{4}D_{P}$$

where *r* is the distance from the planet center to the point on the magnetopause surface described by the angle *θ*, the angle between the position vector of this point and the planet‐Sun line. The surface is parameterized in terms of the standoff distance, *r*_0_, which controls the size of the magnetosphere, and the “flaring” parameter, *K*, which controls the downstream shape as shown in Figure 1. As well as the solar wind dynamic pressure, *D*_P_, *Shue et al.* [1997] also presented forms of the magnetospheric standoff distance and the flaring parameter that depend on the orientation of the interplanetary magnetic field (IMF). Dayside magnetic reconnection is most efficient when the IMF and planetary magnetic fields are antiparallel so, as a result, extended periods of southward IMF cause erosion of the dayside magnetopause due to enhanced reconnection [e.g., *Aubry et al.*, 1970].

This model was applied to Saturn's magnetopause by *Arridge et al.* [2006] using observations from the first six orbits of the Cassini spacecraft, along with the flybys of Voyager 1 and 2. The coefficients *a*_i_ were determined using an interior reflective Newton‐Raphson fitting routine to fit the model to a set of Cassini observations. Coefficients *a*_1_ and *a*_2_ set the size scale and the compressibility, or response to changes in *D*_P_, of the system. The IMF dependency was omitted because it could not be measured in the absence of a dedicated upstream monitor close to Saturn. More recently, MHD simulations by *Jia et al.* [2012] have found that the magnetosphere is insensitive to changes in the IMF.

In the absence of a dedicated upstream dynamic pressure monitor, *D*_P_ was calculated by balancing its normal projection with the interior magnetic pressure adjacent to the magnetopause current layer. A Newtonian pressure balance equation from aerodynamic studies of supersonic flow around a body was used, which was first applied in the context of magnetospheric physics by *Petrinec and Russell* [1997]:

(4) $$\frac{B^{2}}{2\mu_{0}}=kD_{P}\overset{2}{\cos}\Psi +P_{0}\overset{2}{\sin}\Psi$$

where *B* is the magnitude of the interior magnetic field and *μ*_0_ is the permeability of free space. Ψ is the angle between the direction opposite to the upstream solar wind velocity, assumed to be along the Sun‐planet line, and the normal to the magnetopause surface at the observation location. *P*_0_ is the static (thermal) component of the magnetosheath pressure. The constant factor *k* relates to the divergence of streamlines of flow around the magnetosphere, which acts to reduce the dynamic pressure. In the high Mach number regime appropriate for Saturn [e.g., *Slavin et al.*, 1985], a value of *k* ∼ 0.88 is applicable as shown by *Walker and Russell* [1995]. It can readily be seen that close to the standoff point, where Ψ→0°, the dynamic pressure dominates, but away from the “nose” of the magnetosphere, where Ψ→90°, the dynamic pressure term reduces to zero and static pressure dominates in the lobes.

*Kanani et al.* [2010] used the same empirical model but improved on previous studies by using more magnetopause observations. They also used measurements from the Magnetospheric Imaging Instrument (MIMI) [*Krimigis et al.*, 2004] and the Electron Spectrometer (CAPS‐ELS) [*Young et al.*, 2004] to estimate the suprathermal magnetospheric plasma and electron pressures respectively. Together with the magnetic pressure and the assumption of pressure balance across the magnetopause, a more realistic estimate of the dynamic pressure was obtained. In addition, *P*_0_ was expressed as a function of *D*_P_ as previous estimates were too small to be consistent with MHD simulations. Furthermore, if *P*_0_ is kept constant but exceeds a critical value, imaginary flow velocities are introduced.

As a result, *Kanani et al.* [2010] proposed the following modified pressure balance condition across the magnetopause boundary,

(5) $$kD_{P}\overset{2}{\cos}(\Psi )+\frac{k_{b}T_{\text{SW}}}{1.16m_{p}u_{\text{SW}}^{2}}D_{P}\overset{2}{\sin}(\Psi )=\frac{B^{2}}{2\mu_{0}}+P_{\text{MIMI}}+P_{\text{ELS}}$$

where *k*_*b*_ is the Boltzmann constant, *m*_*p*_ is the mass of a proton, *T*_SW_ and *u*_SW_ are the solar wind temperature and velocity respectively, for which values of 100 eV [*Richardson*, 2002] and 460 km *s* ^−1^ have been assumed for the present study, *P*_MIMI_ is the pressure contribution of suprathermal water group ions (see *Sergis et al.* [2009] for details] and *P*_ELS_ is the thermal electron pressure contribution. The constant factor 1.16 accounts for a 4% density abundance of He^+^ in the solar wind with a temperature approximately 4 times greater than that of the protons [*Robbins et al.*, 1970]. *Kanani et al.* [2010] found that the dynamic pressure is insensitive to the values assumed for *T*_SW_ and *u*_SW_ since the second term of equation (5) is much smaller than the first term for any reasonable values of solar wind parameters and for almost the full range of Ψ.

The empirical model described above is capable of representing both open and closed magnetospheres but is axisymmetric about the planet‐Sun line. *Lin et al.* [2010] modified this functional form to allow both north‐south and east‐west asymmetries and fitted it to magnetopause crossings from many different spacecraft in orbit around the Earth using a Levenberg‐Marquardt solver in several stages. Even with these modifications, they found that, globally, the Earth's magnetopause is largely axisymmetric at equinox but the local structure changes substantially in the cusp regions.

The physical properties of Saturn and Jupiter and their magnetospheres relative to the Earth (e.g., high rotation speeds, internal plasma sources, and magnetospheric size scales) imply that the internal dynamics taking place at these systems are significantly different to those within the Earth's magnetosphere. It follows that the geometry of Saturn's magnetopause is likely to be significantly different to the terrestrial magnetopause. *Pilkington et al.* [2014] explored the high‐latitude structure of the Kronian magnetopause using the first set of highly inclined orbits of Cassini from 2007 to 2009. They also used equation (5) to estimate the dynamic pressure but omitted the thermal electron pressure moments as *Kanani et al.* [2010] found that they were, on average, 2 orders of magnitude smaller than the suprathermal ion pressure moments and, hence, negligible in this context. *Pilkington et al.* [2014] also considered the pressure contribution associated with the centrifugal force at the magnetopause using the magnetodisc model of *Achilleos et al.* [2010a] but found that this was also negligible compared to other contributions represented in equation (5).

After identifying a departure between the observed locations and the locations predicted by the axisymmetric magnetopause model, *Pilkington et al.* [2014] modified the model in order to incorporate polar confinement by applying a simple dilation along the *Z*_KSM_ axis by a factor E. They used high‐latitude magnetopause crossings to determine which value of E provided the most statistically significant fit to this data and consequently found that flattening the surface by ∼19% along the *Z*_KSM_ axis provided the best fit. Their data, however, were restricted to the northern hemisphere on the duskside of the planet. In addition, *Kivelson and Jia* [2014] studied the Kronian magnetosphere using MHD simulations and identified a dawn‐dusk asymmetry in the average extent of the magnetopause. This is not incorporated into the current work but will be the subject of a future study.

### 2.2 This Study

In this study, the empirical surface described by equations (1), (2), (3) is modified by incorporating polar flattening by simply reducing the extent of the magnetopause along the north‐south direction by a scaling factor E as done by *Pilkington et al.* [2014]. This is included as a free parameter when fitting this surface to the set of data described in section 3. The ultimate aim is to determine the set of coefficients *a*_*i*_ and E that minimize the distance between the observed magnetopause and the location predicted by the model for each magnetopause crossing. After calculating the crossing‐surface distance for each crossing using the method described in Appendix B, the root‐mean‐squared (RMS) residual is calculated and is minimized until it reaches a tolerance of 10^−6^ *R*_*S*_.

The first stage of this procedure involves estimating the dynamic pressure at the time of each magnetopause crossing, at which the model surface will be constructed. The same method is employed to estimate the dynamic pressure as in the studies described above, but we also estimate the pressure contribution from the water group ion population with energies <45 keV (which we define as “low energy”). *Kanani et al.* [2010] accounted for the pressure associated with low‐energy protons by assuming that their number density is 20% of the low‐energy electron density and, hence, that they have a pressure contribution equivalent to 20% of the electron pressure assuming equal temperatures. However, the pressure associated with the water group ions within this energy range was not included by *Kanani et al.* [2010].

*Thomsen et al.* [2010] surveyed the properties of the low‐energy ion population using the CAPS ion mass spectrometer. They found that beyond *L* ∼ 11*R*_*S*_ the pressure associated with the thermal water group ion population at the rotational equator is comparable to the suprathermal contribution, in agreement with the results of *Sergis et al.* [2010]. To obtain an upper limit estimate of the additional contribution made to the magnetospheric pressure by the thermal ions, we make use of the same data as used by *Thomsen et al.* [2010] with equatorial pressures binned by *L*. But instead of the bin averages [cf *Thomsen et al.*, 2010, Figure 12], the maximum pressures found in each bin are fitted to. The resulting upper limit profile is given by,

(6) $$P_{e}(\text{n Pa})=287L^{-3.14}$$

where *P*_*e*_ is the equatorial pressure measured in nanopascal at the center of the plasma sheet and *L* is the distance between the planet center and the equatorial crossing of the dipole field line that passes through the point of interest.

The energy ranges of the CAPS and MIMI instruments overlap between 3 and 45  keV, and pressure moments derived from the latter are also used in this study, meaning that the pressure contribution for ions in the overlap region may be counted twice. However, *Sergis et al.* [2010] found that the overestimation of the total pressure due to this overlap is generally less than 25% as the sensitivity of the CAPS instrument drops as it approaches its upper limit detection threshold. This is small compared to the scatter in the data.

To account for the strong centrifugal confinement of the thermal plasma near the current sheet, the equatorial pressure (equation (6)) is scaled with height above the spin equator, *z*, in the same way as *Hill and Michel* [1976],

(7) $$P_{\text{cold}}(z)=P_{e}\;\exp\left(-\frac{z^{2}}{H^{2}}\right)$$

where *H* is the ion‐scale height at Saturn's magnetopause, which was found to be ∼5*R*_*S*_ for W^+^ at *L* ∼ 17 [*Thomsen et al.*, 2010].

*Arridge et al.* [2008, 2011] found that the plasma sheet is deflected out of the spin equator as a function of planetary season due to solar wind forcing and that it oscillates about this mean position in phase with the magnetic oscillation [e.g., *Andrews et al.*, 2008]. To determine the effective value of *z* in equation (7) for each of the magnetopause crossings in our study, we reference the spacecraft position with respect to the expected location of the current sheet, given by *Arridge et al.* [2011]:

(8) $$z_{\text{CS}}=\left[\rho -r_{H}\tanh\left(\frac{\rho }{r_{H}}\right)\right]\tan\theta_{\text{SUN}}+(\rho -\rho_{0})\tan\theta_{\text{TILT}}\cos\Psi_{\text{PS}}$$

where *z*_CS_ is the displacement of the current sheet away from the spin equator, *ρ* is the cylindrical distance from Saturn measured in the equatorial plane, *r*_H_ is the characteristic distance where current sheet “hinging” begins and *θ*_SUN_ is the subsolar latitude. *ρ*_0_ is the distance at which the plasma sheet becomes tilted, *θ*_TILT_ is the tilt angle of the plasma sheet and, finally, Ψ_PS_ is the phase of the plasma sheet oscillation.

The hinging distance has been taken to equal the standoff distance of the magnetopause surface that passes directly through each crossing location as suggested by *Arridge et al.* [2008]. Values for *θ*_TILT_ (7.0°) and *ρ*_0_ (10*R*_S_) were chosen in order to maximize the displacement of the oscillating current sheet while remaining consistent with the results of *Arridge et al.* [2011]. The current sheet was chosen to be centered on any magnetopause crossing where its combined hinging and oscillation could cause it to move to such a position, thus maximizing *P*_cold_. Hence, equation (5) then becomes

(9) $$kD_{P}\overset{2}{\cos}(\Psi )+\frac{k_{b}T_{\text{SW}}}{1.16m_{p}u_{\text{SW}}^{2}}D_{P}\overset{2}{\sin}(\Psi )=\frac{B^{2}}{2\mu_{0}}+P_{\text{MIMI}}+P_{\text{ELS}}+P_{\text{cold}}$$

The upper limit *P*_cold_ that is used here is comparable to but smaller than *P*_MIMI_, in general, but *P*_cold_/*P*_MIMI_≪1 for the high‐latitude crossings as anticipated. Including the *P*_cold_ term provides a small improvement to the fitting RMS residual, but the parameters derived from fitting the empirical model to the data set described in section 3 are insensitive to its inclusion (within the fitting uncertainties at the 2*σ* level).

## 3 In Situ Magnetopause Observations

The *Kanani et al.* [2010] study covered magnetopause crossings of the Cassini spacecraft from before Saturn Orbit Insertion (SOI, July 2004) up until January 2006, during which time the spacecraft sampled the low‐latitude magnetopause up to ∼40*R*_S_ beyond the terminator on the dawn side of the planet. *Pilkington et al.* [2014] covered from early 2007 to the end of 2008, during which the spacecraft sampled the high‐latitude magnetopause in the northern hemisphere on the duskside of the planet but had far poorer coverage of the equatorial magnetopause. We have reidentified crossings during the interval covered by *Kanani et al.* [2010] and, in general, find very good agreement with the original analysis.

**Figure 2:**
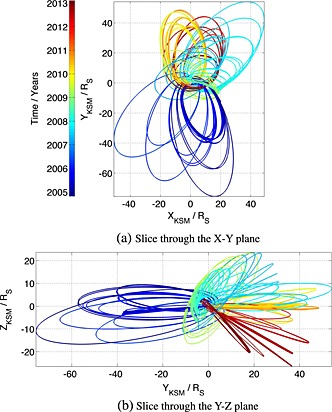
Spacecraft trajectories containing at least one crossing of the magnetopause from just prior to SOI in 2004 to late 2010 and from mid‐2012 to early 2013.

The present study utilizes the data covered by *Kanani et al.* [2010] and *Pilkington et al.* [2014] and extends it such that crossings from 28 June 2004 (just prior to SOI) to 29 October 2010 and from 13 May 2012 to 8 February 2013 is covered. The latter period was added because the high‐latitude magnetopause in the southern hemisphere was sampled during this time, and coverage was extended from the conclusion of the *Pilkington et al.* [2014] study to late 2010 in order to attain better coverage of the equatorial magnetopause on the dawn side of the planet. These trajectories are shown in Figure 2. *Pilkington et al.* [2014] analyzed the trajectory of the spacecraft to ensure they had adequate sampling, such that their results were not biased by observations of extreme magnetopause configurations. That exercise is not repeated herein, but their results are used in order to reduce the data to avoid bias where necessary. Specifically, *Pilkington et al.* [2014] found that they had good sampling of the high‐latitude magnetopause for *X*_KSM_ ≥ 2.5*R*_*S*_.

It should also be noted that since this study spans a sizeable fraction of a Kronian year, seasonal variability in the magnetopause geometry is now an issue of which to be wary. Specifically, *Maurice et al.* [1996] and *Hansen et al.* [2005] found a significant north‐south asymmetry in the magnetopause geometry under conditions where the magnetic dipole is not orthogonal to the direction of solar wind flow. Such a situation occurs over the majority of the Kronian year and they are only truly orthogonal at equinox. This is thus expected to affect the location of the high‐latitude magnetopause crossings. However, in the current study, all high‐latitude observations were made at similar hemispheric season since the crossings in the northern and southern hemispheres were separated by roughly 6 years. The magnetic dipole was titled away from the Sun by ∼10–14° in the northern hemisphere in 2007 when the high‐latitude observations were made in that region. Similarly, in the southern hemisphere the dipole was tilted away from the Sun by ∼14‐17° in 2012‐2013 when the high‐latitude observations took place there. As such, one may expect the degree of polar flattening to be similar in both hemispheres. Indeed, if the empirical model outlined in section 2.2 is fitted to crossings in each hemisphere separately as outlined in Appendix A, the same degree of polar flattening is retrieved within the fitting uncertainties. It is thus assumed for this particular data set that it is appropriate to fit a single empirical model describing polar flattening using a single free parameter. However, this effect will be further quantified in a future study.

**Figure 3:**
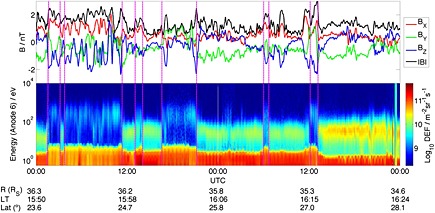
Several magnetopause crossings (magenta vertical lines) identified using (top) MAG and (bottom) CAPS‐ELS data. When the spacecraft passes from the magnetosheath to the magnetosphere, these are typically characterized by a sharp increase in the field strength and usually a rotation in the field components. The field is usually much more variable in the magnetosheath too. The MAG data shown here has a resolution of 1 min but is smoothed using a moving average filter with a span of 11 min. A sharp decrease in the electron count rate (proportional to density) is also observed in addition to a sharp increase in the average electron energy.

Data from the Cassini Fluxgate Magnetometer (MAG) [*Dougherty et al.*, 2002] and CAPS‐ELS were used to identify magnetopause crossings. Some of these observations are shown in Figure 3. The internal pressure was estimated for each crossing by summing field and plasma pressures just inside the magnetosphere, averaged over a time interval no smaller than 20 min in duration, as they can be highly variable. In total, 1607 magnetopause crossings were identified. Of these, MIMI pressure moments were unavailable for 93, leaving 1514.

In previous studies, crossings closely separated spatially and in time were averaged together to prevent artificial weighting due to boundary motions [e.g., *Slavin et al.*, 1983; *Arridge et al.*, 2006; *Kanani et al.*, 2010]. We point out here that due to the underlying assumption of pressure balance across the magnetopause, this practice could, in fact, be detrimental to the study and could reduce the accuracy of the model. This is because the magnetopause moves much faster than the spacecraft (to zeroth order, it can be assumed that the spacecraft is stationary with respect to the magnetopause). As a result, if the magnetopause is observed on multiple occasions within a short period of time, it is likely to be close to equilibrium because, otherwise, it would be observed just once as it moves rapidly past the spacecraft. So, in that sense, not performing this averaging could improve the study as, essentially, measurements where pressure equilibrium is a good assumption would be (slightly) more highly weighted. Furthermore, *Jia et al.* [2012] found using MHD simulations that even under steady solar wind conditions the magnetopause experiences periodic movements. Temporal variability of the magnetopause under such conditions will be preserved by using the full set of data without averaging.

For completeness, the effect of averaging on the results of this study were investigated by averaging crossings on the dawn and dusk sides within 5 and 3 h of each other, respectively, in accordance with the study of Saturn's boundary motions by *Masters et al.* [2012]. In practice, if two crossings were observed within this period the one with the poorer statistics was discarded. In some cases, the estimated dynamic pressure between the two observations was significantly different, which added additional scatter to the data when the quantities were averaged. Since such crossings are close together both temporally and spatially, averaging their positions makes very little difference to their locations. After averaging, 737 crossings remained with which to fit the model. It was found that averaging had no significant effect on the fitting results presented in later sections, so the results fitted to the entire data set without averaging (i.e., 1514 crossings) are presented. The only difference between the two methods was the magnitude of the uncertainties in the fitted model parameters, which, of course, are smaller when the full data set is used.

**Figure 4:**
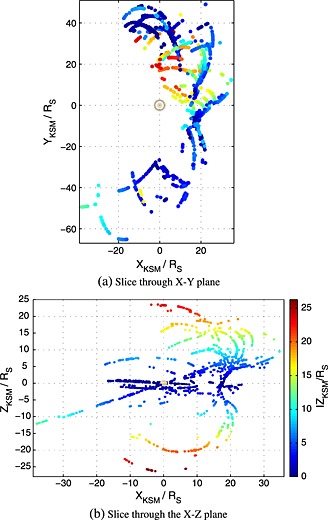
The distribution of the magnetopause crossings between SOI in 2004 to late 2010, and from mid‐2012 to early 2013 in the KSM system colored by their *Z*_KSM_ coordinate with the planet at the origin. (a) There is good coverage of the equatorial magnetopause on both flanks but high‐latitude coverage is restricted to the dusk flank. (b) That there is high‐latitude coverage of both hemispheres.

The full data set is displayed in Figure 4 in the Kronocentric Solar Magnetospheric (KSM) system, where the *X*_KSM_ axis is along the planet‐Sun line directed toward the Sun, the *Z*_KSM_ axis is oriented such that the planetary magnetic dipole lies within the *X*_KSM_−*Z*_KSM_ plane, and the *Y*_KSM_ axis completes the right‐handed set and is, hence, directed from dawn to dusk. The spacecraft positions were calculated using the reconstructed trajectory kernels of NASA's Navigation and Ancillary Information Facility “SPICE” geometry information system.

## 4 The Impact of *β* on Magnetopause Location

### 4.1 Initial Results

**Figure 5:**
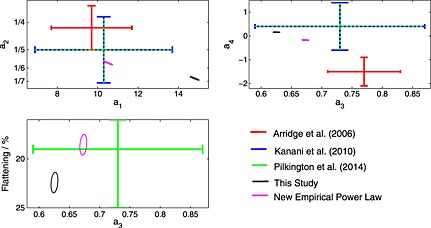
Shows the coefficients obtained by fitting magnetopause surfaces to magnetopause crossing data, all results are displayed at the 2*σ* level. The colored bars are the results of previous studies while the confidence ellipses indicate the result of this study using the usual standoff distance power law (black) and the new *β*‐dependent power law (magenta). See Table 1 for precise values of the coefficients. Note that the uncertainties are much smaller in this study due to the improvements made in the fitting procedure and the large amount of data used.

The initial results of fitting the model to all crossings simultaneously are shown in Figure 5 as the black confidence ellipses, along with the results of previous studies, all at the 2*σ* level. The technical details regarding the fitting methodology and a significant improvement made over previous studies are detailed in Appendices A and B, respectively. The uncertainties have been estimated in two different ways as discussed in Appendix C, though both methods give similar results in this case. Most coefficients are in agreement with previous studies within the fitting uncertainties, but for the coefficients *a*_1_ and *a*_2_ there is a significant disagreement with previous studies. Coefficient *a*_1_ defines the scale size of the system and *a*_2_ defines the compressibility of the magnetosphere—how strongly it reacts to variations in dynamic pressure. We found that *a*_2_ = 1/(7.8 ± 0.4), which apparently indicates that the magnetosphere is very “stiff” and relatively unresponsive to changes in dynamic pressure. A value of one sixth is expected for a dipole magnetic field and is usually considered appropriate in the case of the Earth [e.g., *Shue et al.*, 1997]. A value larger than this is expected for plasma‐laden systems such as those of Saturn and Jupiter. For example, *Kanani et al.* [2010] find *a*_2_ = 1/(5.0 ± 0.8) for Saturn and *Huddleston et al.* [1998] find *a*_2_ = 1/(4.5 ± 0.8) for Jupiter. In this context, our value does not seem physically feasible, at least when predicting the nominal response of the magnetosphere to changes in dynamic pressure.

A slightly different approach to obtaining both *a*_1_ and *a*_2_ is to take the logarithm of equation (2) and rearrange this to form a linear relationship,

(10) $$\log r_{0}=-a_{2}\log D_{P}+\log a_{1}$$

where *D*_P_ is estimated assuming pressure balance as usual and *r*_0_ can be found for each crossing by fitting the surface directly through each crossing. Coefficients *a*_1_ and *a*_2_ can then be obtained from the resulting line of best fit when these quantities are plotted.

The subtle difference between this method and the global fitting method is that in this case, the surface passes directly through each magnetopause crossing. In the previous method, the surface was constructed at *D*_P_, and did not necessary pass directly through each magnetopause crossing. In fact, it is the distance between the surface constructed at *D*_P_ and the crossing location that is used to assess how well the model fits the data as described in Appendix B.

**Figure 6:**
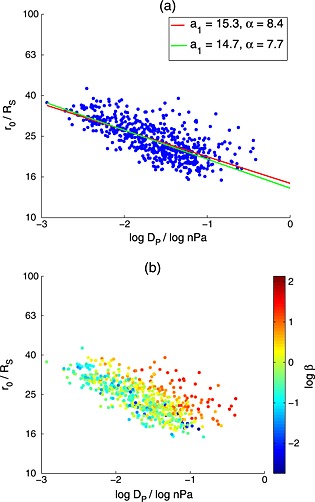
Equation (10) plotted for the crossings used in this study. (a) Two different methods are used to find the coefficients *a*_1_ and *a*_2_ (1/*α*) as described in the text. Within the uncertainties, both methods give the same results and find that *a*_2_ is much smaller than expected for Saturn. (b) The same data with a logβ color scale and shows that the plasma conditions inside the magnetosphere strongly affect the location of the magnetopause.

The results of these two methods of estimating *a*_1_ and *a*_2_ are shown in Figure 6a. Reassuringly, both methods give the same results within the uncertainties. Interestingly though, there appears to be substantial scatter above the main body of the data, whereas there is relatively little below. Figure 6b shows the same data colored by logβ, where *β* is the ratio of the total plasma pressure to the magnetic pressure. This parameter shows a remarkable trend with system size. It shows that the location of the magnetopause is affected dramatically by the plasma conditions adjacent to it, such that the extrapolated standoff location can vary between 10 and 15 *R*_*S*_ between low and high *β* conditions at constant *D*_P_. As such, the standoff distance power law, equation (2), used to model the size of the magnetosphere is not valid as a single one‐dimensional power law cannot account for this variability due to changing *β*. This is much larger than the magnetopause oscillations observed by *Clarke et al.* [2010], typically of amplitude ∼1.2*R*_*S*_ but occasionally as large as ∼4–5 *R*_*S*_. However, a similar degree of variability in standoff location has been identified under low (<0.005 nPa) solar wind dynamic pressure conditions by *Jia et al.* [2012] using MHD simulations.

**Figure 7:**
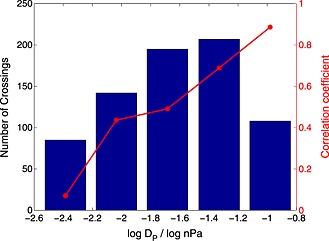
The crossings have been separated into bins of $\log D_{P}$ and the correlation between *β* and *r*_0_ has been calculated. In all cases, this correlation is positive and seems to increase with *D*_P_. Besides the smallest *D*_P_ bin, the *p* value (the probability of such a correlation occurring by chance) is negligible. These correlation coefficients should be taken as lower limits as the *D*_P_ bins are fairly coarse to ensure a representative number of crossings fall within each one.

Furthermore, Figure 7 shows that *β* and *r*_0_ are strongly correlated, and this correlation increases with *D*_P_. There is only a very weak correlation between these quantities within the smallest dynamic pressure group as there are very few high *β* crossings within this group (Figure 6b). A possible explanation for this is that the Cassini orbit usually lies inside the magnetopause when it is greatly expanded, so crossings under conditions of high interior *β* and low dynamic pressure frequently cannot be measured; hence, the correlation between *β* and *r*_0_ is low in these situations. Essentially, a detection threshold is reached whereby the magnetopause can only be sampled when it has a standoff distance smaller than 40 *R*_*S*_ as such a magnetosphere could easily extend to 90 *R*_*S*_ in the terminator plane.

A similar trend is evident between *r*_0_ and the total plasma pressure, though it is weaker than the aforementioned trend between *r*_0_ and *β*. The most likely reason for this is that if the magnetic field is strong enough, it can suppress the expansion of the system since the plasma pressure must be strong enough to change the magnetic field configuration since they are frozen together. The *β* parameter, on the other hand, describes what is controlling the system—is the magnetic field sufficient to confine the plasma or is the plasma pressure strong enough that it can reshape the system and significantly perturb the magnetic field?

**Figure 8:**
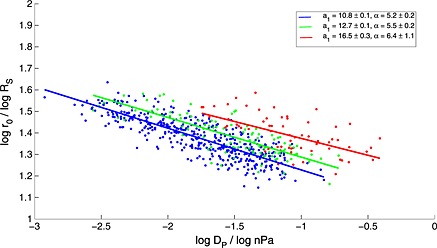
A k‐means clustering algorithm has been used to separate crossings into three clusters based on *β*. They have been split into groups based upon the plasma conditions prevalent at the time of the crossing. Lines of best fit have been fitted through these groups separately. Of particular note is that the factor that governs the size scale of the magnetosphere (*a*_1_) increases with *β* well outside of the uncertainties. This result is insensitive to the number of clusters into which the data are separated (the analysis has been attempted with up to seven).

**Figure 9:**
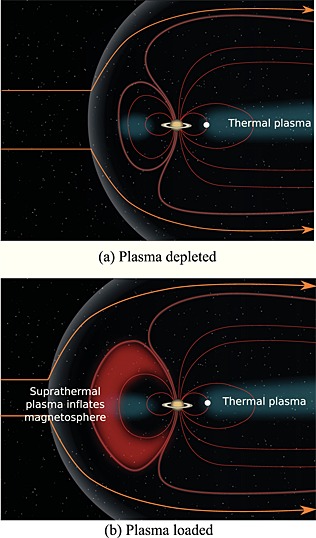
A schematic depicting two snapshots of the system under conditions in which the interior plasma pressure adjacent to the magnetopause is (a) low and (b) high, and the corresponding effects these conditions have on the magnetopause location. When *β* is high, the plasma pressure dominates over the magnetic pressure and can change the magnetic field structure and push out the boundary. The white point indicates Enceladus, a large plasma source within the system. The magnetic field lines are distended radially outward when the hot plasma pressure is increased in the corresponding force balance within the magnetosphere [*Achilleos et al.*, 2010b].

In the first instance, one could repeat the analysis over small intervals of *β* to identify how the system scales under different internal conditions. There are many different methods one could use to split the data; here, a k‐means clustering algorithm is used to separate the data as naturally as possible but in reality *β* is continuous and any small interval of *β* could be chosen provided that it contains enough crossings. This algorithm has been used to separate the data into three intervals of *β* and separate best fit lines were fitted through each cluster as shown in Figure 8. In each case, the magnetospheric compressibility remained the same within the estimated uncertainties and was 1/(5.5 ± 0.2) on average, in agreement with *Kanani et al.* [2010]. However, *a*_1_, which scales the size of the magnetosphere, changed between clusters well outside of the uncertainties and in the same sense as the average value of *β* for each cluster. This indicates that the magnetosphere can exist in a relatively plasma‐depleted or plasma‐loaded state as indicated schematically in Figure 9.

Conceptually, this makes sense. Consider the simplified situation where the magnetosphere is initially in steady state such that the internal and external pressures are equal. If the interior plasma pressure then increases, the instantaneous *β* will also increase and the magnetosphere will expand in order to reestablish equilibrium. Hence, even for a steady dynamic pressure there is a range of plausible standoff distances depending on the internal conditions as a result of gradual mass loading. The large fluctuations in the observed interior plasma conditions may be caused by quantized plasmoid loss as a result of Vasyliũnas‐style reconnection in the magnetotail and the resulting planetward flow of energized plasma [*Vasyliunas*, 1983], or through interchange events as observed via energetic neutral atom imaging by the Ion and Neutral Camera on board Cassini [e.g., *Krimigis et al.*, 2007; *Brandt et al.*, 2010; *Mitchell et al.*, 2015]. Both of these types of events lead to rapid changes in the interior plasma pressure and are expected to affect pressure balance at the magnetopause boundary as a result.

The usual standoff distance power law cannot account for these effects as the scaling factor, *a*_1_, must change to compensate for the resulting change in the geometry of the magnetopause even while the solar wind pressure remains steady. What is unclear, however, is how *β* changes as the system expands. Ultimately it depends on the rate of change of the magnetic field strength and the plasma pressure with respect to system size. A theoretical treatment of this process could be the subject of future work.

### 4.2 Incorporating *β* Into the Empirical Magnetopause Model

The original standoff distance power law was derived in the context of the Earth's magnetosphere, which is relatively devoid of plasma at the magnetopause boundary. As such, it is a good approximation to assume that the solar wind dynamic pressure is balanced by the magnetic pressure alone.

Of course, this is far from true of the magnetospheres of Saturn and Jupiter and will be addressed here. The dynamic pressure at the standoff point can be approximated as

(11) $$D_{P}\propto \frac{B^{2}}{2\mu_{0}}(1+\beta )$$

At this location, the magnetic field at the standoff point can be expressed as $B=B_{0}r_{0}^{-1/2a_{2}}$, where *B*_0_ is the equatorial magnetic field at the surface of the planet. This power law is valid over a wide range of standoff distance as found by *Bunce et al.* [2007] and *Achilleos et al.* [2014] but is affected by the magnetospheric plasma content, which causes *a*_2_ to change. Hence, *r*_0_ can be expressed as

(12) $$r_{0}=a_{1}{\left(\frac{D_{P}}{1+\beta }\right)}^{-a_{2}}$$

Note that strictly speaking, *β* in equation (12) should be the plasma *β* measured just inside the standoff point. In the absence of this information, the locally measured *β* will be used in the first instance. The results of repeating the fitting procedure with the new standoff distance power law are shown in Figure 5 by the magenta ellipses at the 2*σ* level. Now, all results are in agreement with previous studies and incorporating *β* into the empirical model results in a decrease in the RMS residual by 0.8*R*_*S*_, indicating a large increase in the accuracy of the model without the use of additional free parameters.

In the first instance, this may appear puzzling because earlier analyses did not include the *β* dependence, so they should agree better with the analysis performed without *β*? The explanation for this apparent paradox is that the data used in these earlier studies were confined to a region of the magnetosphere where *β* is, in general, relatively small. The *β* dependence is still present within these data but has a much smaller influence. For these data, the median *β*1.6 in comparison with *β*∼3.0 across the entire data set.

The fact that using the local value leads to such a large increase in the predictive power of the model indicates that there may be a strong correlation between the local and nose *β*. Indeed, adding an additional free parameter to scale the local *β* to that expected at the standoff point improves the accuracy of the model by 0.3*R*_*S*_ with a scale factor of ∼0.4. However, after performing a *F* test on these models, it was found that the additional free parameter does not provide a statistically significant improvement to the predictive power of the model so will not be discussed further.

The magnetospheric compressibility now agrees with previous studies though is more “Earth‐like” (more dipolar) than previous studies. In addition, it was found that the dynamic pressure has only a very small effect on the magnetospheric flaring, so can be safely neglected in future studies with minimal loss of model accuracy.

### 4.3 Revisiting Bimodality

*Achilleos et al.* [2008] used magnetopause crossings observed between 1 July 2004 and 3 September 2005 to assess the long‐term statistical behavior of Saturn's magnetosphere. They reported that the magnetospheric standoff distance, which is a proxy for the global size of the magnetosphere, exhibits a bimodal structure, meaning that there are two most likely standoff distances associated with the internal magnetospheric configuration. It is plausible that these “modes” correspond to measurements in which the magnetopause is caught in either a plasma‐loaded or a plasma‐depleted state with a relatively rapid transition between these states.

This study is an ideal opportunity to revisit bimodality in light of the much larger data set that has been amassed. The standoff distance has been calculated for each magnetopause crossing by fitting the best fitting model described in Table 1 directly through each magnetopause crossing. This tends to be a more stable way of calculating the standoff distance than using equations (2) or (12) since information about all of the coefficients is used, and correlations between the coefficients mean that the standoff distances do not change much within the coefficient uncertainties.

**Table 1:** The Fitting Results of the Present Study Are Displayed, Along With the Results of Previous Studies^a^

Parameter	A06	K10	P14	New Model
*a*_1_	9.7 ± 1.0	10.3 ± 1.7		10.5 ± 0.2
*α* = 1/*a*_2_	4.3 ± 0.3	5.0 ± 0.8		5.7 ± 0.1
*a*_3_	0.77 ± 0.03	0.73 ± 0.07		0.67 ± 0.01
*a*_4_	−1.5 ± 0.3	0.4 ± 0.5		0.17 ± 0.03
Flattening %			19 ± 3	19 ± 1
RMS when applied to new data	7.35	4.70 (4.98)^b^	4.60 (3.51)^b^	3.54 (3.13)^b^
No. of data points (No. averaged)	64 (26)	191 (68)	626 (196)	1514

a The RMS residual found between each model and the new data set is shown to indicate the goodness of each fit.

b For high‐latitude crossings (≥30°) A06, K10, and P14 are the empirical models of *Arridge et al.* [2006], *Kanani et al.* [2010], and *Pilkington et al.* [2014], respectively.

**Figure 10:**
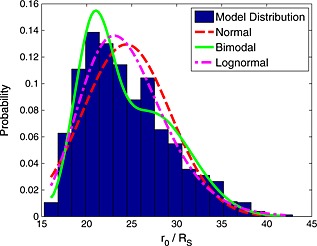
The distribution of extrapolated standoff distances found by fitting the best fitting model specified in Table 1 through the precise location of each magnetopause crossing. On top of this are plotted normal (red, dashed line), bimodal (green, solid line), and lognormal (magenta, dash‐dotted) distributions fitted to these data.

Figure 10 shows a histogram of empirical standoff distance with normal, lognormal (the best fitting example of a skewed distribution in this case), and bimodal distributions fitted to the data. Statistical tests have been used to determine which of these provides the best fit to the data. First of all, the Kolmogorov‐Smirnov test [*Massey*, 1951] has been applied to test the null hypothesis that the data could have arisen from an underlying population that follows each distribution. Using this test, the normal distribution was overwhelmingly rejected with a negligible *p* value, which can be interpreted as the probability of obtaining a distribution at least as extreme as that observed provided that the null hypothesis is true. Since this probability is negligible, this test implies that the underlying standoff distance population is very unlikely to be normally distributed. The *p* value was much larger for the lognormal distribution but was still negligible (approximately one in a million). On the other hand, the *p* value for the bimodal distribution is ∼0.17. While this probability is still fairly low, it shows that the bimodal distribution is far more likely to describe the underlying population from which our data are drawn. Even so, the low probability indicates that the bimodal distribution is not able to capture the behavior of the magnetopause entirely. It is possible that the degree of skewness evident in the distribution could be the reason why the *p* value is still quite small for the bimodal distribution.

Higher‐order distributions can also be tested, such as a “trimodal” distribution, which yields a *p* value of 0.54. However, care must be taken not to overfit: the *p* value will asymptotically approach 1 as more free parameters are added to the model. To ascertain is this is the case, the Bayesian information criterion (BIC) [*Schwarz*, 1978] can be calculated. This is a measure of the information retained by the model while penalizing additional free parameters. The model that minimizes the BIC is the model that retains the most information about the distribution without introducing extraneous free parameters. In this case, the model that achieves this is a bimodal distribution with means at 20.7 and 27.1 *R*_*S*_ with a mixing proportion of 43% and 57%, respectively.

Previous analyses by *Joy et al.* [2002] and *Achilleos et al.* [2008] found that such bimodal behavior could not be explained by the solar wind alone. This is supported by *Jackman et al.* [2011] who analyzed the solar wind conditions upstream of Saturn and Jupiter and found that the dynamic pressure distribution was best described by a single peak. This implies that the second peak in the distribution may be caused by internally driven plasma dynamics. Specifically, it may be symptomatic of the cycle of mass loading and unloading described by *Vasyliunas* [1983]. If the transition betweenthe loaded and unloaded states is rapid compared to the time that the system actually spends in each state, it stands to reason that the magnetosphere would be observed less often in this intermediate state.

## 5 Summary: A Global Magnetopause Model

Here the largest and most complete set of Kronian magnetopause crossings to date has been assembled, covering ∼7 years of the Cassini mission and sampling far more of the global surface geometry than ever before. Assuming balance between pressure sources internal and external to the magnetosphere, the solar wind dynamic pressure has been estimated and a pressure‐dependent surface was fitted to the location of these crossings.

Several key modifications were made to the fitting procedure over previous studies. First, a more sophisticated solver was used to explore parameter space efficiently and ensure that the set of parameters that correspond to the global minimum are found. Second, the distance between each magnetopause crossing and the empirical surface was calculated exactly. It was found that this made a significant difference to the degree of magnetopause flaring and smaller differences in the other parameters compared to the approximate method used in previous studies, which led to an increase in the accuracy of the model by ∼0.6*R*_*S*_. Finally, the thermal ion pressure contribution was calculated more rigorously by exploiting the results of previous work and resulted in an additional increase in the model accuracy.

The dynamic pressure alone was not enough to account for the variability in the size of the magnetosphere and, furthermore, the extra variability could be attributed to dynamic plasma processes inside the magnetosphere, which can cause the magnetosphere to expand by 10–15 *R*_*S*_ at constant *D*_P_. This is much larger than the periodic oscillation of the magnetopause location with amplitude ∼1.2 *R*_*S*_ as found by *Clarke et al.* [2010] but is consistent with MHD simulations which exhibit a similar degree variability under low solar wind dynamic pressure conditions [e.g., *Jia et al.*, 2012]. This internal variability could be characterized in terms of the plasma *β* just inside the magnetopause and, subsequently, this effect was incorporated into the global fitting routine by adding a *β* dependency to the power law used in previous studies that relates the size of the magnetosphere to the dynamic pressure. This results in a substantial increase in the accuracy of the model's predictions, reducing the RMS residual by ∼0.8*R*_*S*_ (the model coefficients are displayed in Table 1). The internal variability described here may be associated with the build up and subsequent loss of plasma from the system and may explain why the sizes of Jupiter's and Saturn's magnetospheres exhibit bimodality as found by *Joy et al.* [2002] and *Achilleos et al.* [2008], respectively, and supported by these observations.

In a future paper, we will attempt to resolve significant asymmetries in the structure of the magnetopause. Further studies should also look at more complex magnetopause structures, such as cusp indentation regions. *Maurice et al.* [1996] found that Saturn's magnetopause has significant cusp indentation regions that could be implemented into future empirical models as was done by *Lin et al.* [2010] to describe the terrestrial magnetopause. The main barrier to this is a lack of cusp crossings to constrain such a model. During the course of this study, ∼10 magnetopause crossings were identified within the cusp region, identified as such due to their high‐latitude location and very low *β*.

Finally, *Clarke et al.* [2006] observed smaller‐scale oscillations in the location of the boundary caused by oscillations in the magnetic field and plasma signatures that are known to occur throughout the Kronian system. Similarly, *Zieger et al.* [2010] found that the periodic release of plasmoids down into the magnetotail causes the magnetopause to oscillate as the resulting waves propagate through the system. For the present study these are neglected but could be added to the existing model as an extra layer of complexity on top of the internally driven variability already discussed.

## Supporting information

Data Set S1

## Figures and Tables

**Figure B1:**
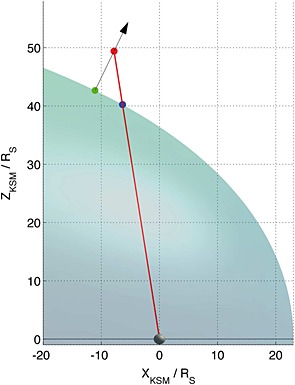
This figure illustrates how the distance between a magnetopause crossing and the empirical surface described by *a*_*i*_ is calculated. The red point is the observed magnetopause crossing, the shaded region is the empirical surface constructed at *D*_P_ and the planet is shown at the origin. The blue point is where the crossing radial vector (red line) intersects the surface and the green point is the closest point on the surface to the crossing, which is found iteratively. A view of the plane containing all three points of interest is displayed. The arrow indicates the normal to the surface at the closest point to the crossing and shows that it passes directly through the crossing location, meaning that the green point is, indeed, the closest point.

## Appendix A Empirical Model Fitting

### A.1

An iterative method is used to fit the model surface to observations of the magnetopause starting from an initial set of parameters *a*_*i*_. The fitting routines used by *Arridge et al.* [2006] and *Kanani et al.* [2010] are implementations of the gradient‐based family of Newton‐like solvers which start at a given set of coefficients and iterate until convergence is achieved, guided by the RMS residual. One of their major drawbacks is that they can only achieve convergence within the basin of attraction within which the starting parameters fall, and this may not be the global minimum of all possible solutions. Often, this is mitigated by repeating the fitting at different starting values to have a better chance of finding the global minimum.

Here a more sophisticated algorithm described by *Ugray et al.* [2007] is used which aims to locate the global minimum efficiently by generating a set of trial points which are then ordered based on their feasibility in terms of the fitting constraints. The only constraints used in this study are bounds on the coefficients; this is necessary as it is infeasible to sample all of parameter space to look for the best solution. In most cases, these bounds were chosen at the physical limit for each coefficient. Where there was no physical reason to restrict a parameter, very large bounds were chosen such that the solutions from past studies are contained by at least 3 times the estimated uncertainty. The best fitting coefficients obtained in this study were all also more than 3*σ* away from the bounds prescribed.

A local solver is initiated at each trial point in the sequence and a list of these starting points, the solutions that the solver ultimately converged to and the distance between these start and end points is complied. Several different local solvers of varying complexity were experimented with by generating a synthetic set of magnetopause crossings from a known model, preserving the distribution of the quantities measured in situ and, finally, adding Gaussian noise to them. Each solver was then tried on this synthetic data to evaluate which solver was able to most closely replicate the known model. Ultimately, the implementation of the interior point algorithm of *Waltz et al.* [2005] in the MATLAB Optimization Toolbox provided the most accurate results. Between each call, the maximum distance between the trial solutions that successfully converged and the solutions they ultimately converged to is used to estimate the radius of the basin of attraction for subsequent trial solutions by multiplying it by an empirically chosen scale factor. This scale factor is used to balance the compromise between efficiency and accuracy and was found by running the global solver on many different problems of varying complexity.

At each iteration, the model described by parameters *a*_*i*_ was fitted precisely through the location of each observed magnetopause crossing to within a tolerance of 10^−6^ *R*_*S*_ in order to calculate Ψ and hence *D*_P_. Initially, a simple Newton‐Raphson solver was used to do this efficiently, but it was found that successful convergence for many crossings was sensitive to the choice of *a*_*i*_. Now, more sophisticated solvers from the MATLAB Optimization Toolbox are used. A Levenberg‐Marquardt solver is used in the first instance as convergence can be achieved for the vast majority of crossings for any given set of coefficients relatively efficiently using this solver. For crossings where this is not possible, the trust region reflective solver is used, which is more computationally expensive but has yet to fail to converge for this purpose. Complete convergence is necessary because the top level solver requires a smooth objective function and gradient thereof to operate correctly, an assumption which is violated if convergence is not achieved for every single crossing fed into the solver.

## Appendix B A Better “Fitness” Criterion

### B.1

In order for the solver to know in which direction to move in parameter space, a goodness‐of‐fit estimator must be calculated at each iteration. A good choice is the distance between each crossing location and the surface described by *a*_*i*_ and *D*_P_. Previous studies used the distance between the crossing and the point where the model surface intersects the crossing radial vector (the blue and red points in Figure B1, respectively) as an approximation for the distance between the crossing and the model surface. Here a near‐exact solution to the minimum crossing surface distance is found by solving a system of nonlinear equations numerically, using the fact that the shortest distance between a point and a surface is along the normal to the surface that passes through that point,

(B1) $$S(x_{0},y_{0},z_{0})=r_{0}{\left(\frac{2}{1+\cos\theta }\right)}^{K}-r(x,y,z)=0$$

(B2) $$x_{0}-x-Fn_{x}=0$$

where *x* and *x*_0_ are the *X*_KSM_ coordinates of the crossing location and the point on the surface closest to the crossing, respectively, *n*_*x*_ is the *X*_KSM_ component of the outward directed normal to the surface computed at the closest point to the crossing and *F* scales the normal vector to the crossing location and is equal to the distance between the crossing and the model surface if the unit normal is used. Hence, *F* is zero if the model surface passes directly through the crossing location and positive (negative) if the crossing lies outside (inside) the surface. Analogous equations to equation (B2) can be constructed for the other two spatial coordinates, *Y*_KSM_ and *Z*_KSM_. Equation (B1) constrains the solution to lie on the surface, and equation (B2) and its counterparts ensure that the closest point to the crossing is found. An initial guess is required for (*x*_0_,*y*_0_,*z*_0_,*F*), but the ultimate solution does not depend on this guess as there is only one solution that can satisfy these equations for any given magnetopause crossing and *D*_P_. The radial approximation used in previous studies is a good first guess that can be used to minimize computation time.

These equations can be solved for (*x*_0_, *y*_0_, *z*_0_, *F*) for each magnetopause crossing to an arbitrary degree of accuracy and so, effectively, represent an exact solution. The distance between each magnetopause crossing and the model surface, constructed at the dynamic pressure estimated assuming pressure balance from equation (9), was calculated along with the RMS residual. The fitting routine is then iterated until the RMS residual converged to within a tolerance of 10^−6^ *R*_*S*_. Fitting using the radial approximation method applied in previous studies and the exact method presented here yield the same results for all but one of the model coefficients within the estimated uncertainties. The coefficient *a*_3_, which chiefly controls how much the magnetopause “flares” by in equation (3), was significantly different between the two methods. This is because the approximate method breaks down as an estimate of the shortest distance between the crossing and the model surface the further into the tail the model is projected. It is the crossings in this region of space that best define the degree of magnetopause flaring so it stands to reason that *a*_3_ would be the most affected by a change in the calculation of the distance between the crossing and the model surface. The results obtained using both methods were compared and it was found that the new method reduced the RMS residual by ∼0.6*R*_*S*_, indicating a substantial increase in the model accuracy.

## Appendix C Coefficient Uncertainty Estimation

### C.1

The most efficient method of estimating the standard error for each of the model coefficients is to approximate the coefficient covariance matrix. This can be done using the variance in the distance between an observed magnetopause crossings and the location predicted by the model, known as the residual. This distance, *F*, can be calculated using the procedure outlined in Appendix B. The sample variance, *σ* ^2^, can then be calculated as

(C1) $$\sigma^{2}=\frac{1}{N-1}\overset{N}{\underset{j=1}{\sum }}{\left(F_{j}-\mu \right)}^{2}$$

where *N* is the number of observations, *F*_*j*_ is the model‐crossing distance for the *j*th crossing, and *μ* is the sample mean.

The coefficient covariance matrix can then be calculated from the variance and the Jacobian matrix, a matrix of dimensions *i* × *j* of the first‐order derivatives of *F* with respect to coefficient *i*,

(C2) $$J=\left(\begin{array}{ccc} \frac{\partial F_{1}}{\partial a_{1}} & \cdots & \frac{\partial F_{1}}{\partial a_{i}}\\ \vdots & \ddots & \vdots \\ \frac{\partial F_{j}}{\partial a_{1}} & \cdots & \frac{\partial F_{j}}{\partial a_{i}} \end{array}\right)$$

the coefficient covariance matrix, *C*, is then given by,

(C3) $$C={\left(J^{T}J\right)}^{-1}\sigma^{2}$$

where *J* and *σ* ^2^ are evaluated at the best fitting set of coefficients. A first‐order approximation to the standard error of each coefficient can then be determined by taking the square root of the diagonal elements of *C*.

Note, though, that this approach neglects second‐order covariances between the coefficients, which are important if the off‐diagonal elements are comparable to the diagonal elements of *C*. Figure 5 shows that this is, indeed, the case for some of the coefficients used in this study. In particular, Figure 5 (top left) shows that the uncertainty ellipses are significantly inclined which indicates a strong correlation between coefficients *a*_1_ and *a*_2_. The standard error calculated for a particular coefficient using the above procedure can be interpreted as the standard error of one coefficient assuming that the other coefficients remain fixed (the vertical or horizontal extent of the ellipse, depending on the coefficient being considered). Though, if two coefficients are strongly correlated, changing one coefficient tends to cause a change in the other coefficient. Such a correlation indicates redundancy in the model and means that the model can be simplified by expressing one coefficient as a linear law of the other coefficient. In this case, it indicates that system size is strongly linked to the compressibility of the system.

In cases in which a strong correlation is identified, more robust methods can be used to estimate the uncertainties instead. For the purposes of the current discussion, the Monte Carlo Bootstrap method is used. This is a powerful technique that can be used to calculate the distribution of the coefficients with few underlying assumptions, only that the observations are a good representation of the underlying population and that the data are independent. This method involves running the fitting routine many times (400 samplings were used here), fitting the model to a different set of crossings each time. As such, it is computationally expensive.

In this case, *N* magnetopause crossings are selected at random from the full set of *N* crossings, but, crucially, these crossings are selected with replacement. This means that, for a given random sample, some crossings are selected multiple times, whereas some are discarded. As a result, the best fitting coefficients are different for each random sample drawn. These coefficients are recorded and confidence intervals for each coefficient can be evaluated. These confidence intervals can then be corrected for bias and skewness [*Efron*, 1987].

The uncertainties estimated using both methods are comparable, and the maximum of these has been reported for each coefficient in Table 1.
