# Supplementary material for: Internally driven large‐scale changes in the size of Saturn's magnetosphere
Source: J Geophys Res Space Phys. 2015 Sep 10;120(9):7289–306. doi: 10.1002/2015JA021290 (PMC5111417; doi:10.1002/2015JA021290)
Supplement: Supplementary file 1 — Data Set S1 [file JGRA-120-7289-s001.pdf]

AGU PUBLICATIONS

Journal of Geophysical Research: Space Physics

Supporting Information for

Supporting Information for "Internally Driven Large-Scale Changes in the Size of Saturn's Magnetosphere"

N. M. Pilkington[1,2], N. Achilleos[1,2], C. S. Arridge[3], Guio, P.[1,2], A. Masters[4], L. C. Ray[1,2], N. Sergis[5], M. F. Thomsen[6], A. J. Coates[7,2], and M. K. Dougherty[4]

[1]Atmospheric Physics Laboratory, Department of Physics and Astronomy, University College London, London, UK, [2]The Centre for Planetary Sciences, UCL/Birkbeck, London, UK, [3]Physics Department, Lancaster University, Lancaster, UK, [4]Blackett Laboratory, Imperial College London, London, UK, [5]Academy of Athens, Office of Space Research and Technology, Athens, Greece, [6]Planetary Science Institute, Tucson, Arizona, USA, [7]Mullard Space Science Laboratory, Department of Space and Climate Physics, University College London, Dorking, UK

Additional Supporting Information (Files uploaded separately)

Dataset DS1 - list of magnetopause crossings

Introduction

Dataset DS1 contains a list of all of the magnetopause crossings identified through the course of this study. When the spacecraft traverses the magnetopause, it typically samples distinctly different plasma populations and magnetic fields on either side. An example of some magnetopause crossings are shown in Figure~1 of the manuscript, and Section~3 describes how these were determined. The spacecraft positions were calculated using the reconstructed trajectory kernels of NASA's Navigation and Ancillary Information Facility (NAIF) `SPICE' geometry information system. Note that the inbound/outbound determination was not recorded at the time that each crossing was identified, but was instead calculated by post-processing the crossing list. Most determinations appear to be correct, but they should not be relied upon for scientific purposes. Take care especially when there are inconsistencies in the list e.g. an outbound crossing followed by another outbound crossing. Such inconsistencies can occur as a result of e.g. data gaps or boundary waves, but may be the result of an incorrect inbound/outbound determination.

Data Set S1. List of magnetopause crossings. Rows contain individual magnetopause crossings, columns are tab-delimited and contain the following information in order:

YEAR

DOY

HOUR

MINUTE

INBOUND(I)/OUTBOUND(O)

XKSM(R_S)

YKSM(R_S)

ZKSM(R_S)
